# Fiscal deficit in sub-saharan Africa: A new intuition from the institution and political drivers

**DOI:** 10.1371/journal.pone.0291150

**Published:** 2023-09-08

**Authors:** Ezekiel Olamide Abanikanda, James Temitope Dada, Rotimi Ayoade Ogunjumo

**Affiliations:** 1 Economic and Business Policy Department, Nigerian Institute of Social and Economic Research (NISER), Ibadan, Oyo State, Nigeria; 2 Department of Economics, Obafemi Awolowo University, Ile–Ife, Osun State, Nigeria; 3 Department of Economics, Landmark University, Omu-Aran, Kwara State, Nigeria; 4 Landmark University SDG 8 (Decent Work and Economic Growth Group), Landmark University, Omu-Aran, Kwara State, Nigeria; The University of the South Pacific, FIJI

## Abstract

Motivated by the growing fiscal deficits in sub-Saharan Africa, this study examines fiscal deficit’s economic, political, and institutional drivers using a panel of twenty-three sub-Saharan African countries. Panel spatial consistent correlation, dynamic fixed effects autoregressive distributed lag, and feasible generalised ordinary least squares were used as the estimation techniques. Our findings reveal that while per capita income, trade openness, population, and religious tension increase the size of fiscal deficit, bureaucracy quality, government stability, Law and order, and military in politics reduce the extent of fiscal deficit. However, corruption control, democratic accountability, and internal conflict have weaker statistical evidence. Furthermore, the study established evidence of long-run co-integration relationships among institutional factors, economic factors, and fiscal deficits in SSA. Per capita income has a significant positive influence in the short run but a negative effect in the long run. Population and religious tension positively impact fiscal deficit in both periods. However, democratic accountability, government stability, and the military in politics significantly negatively impact fiscal deficit in the long run. This study concludes that beyond economic factors, institutional and political factors are significant drivers of fiscal deficit in sub-Saharan Africa. Therefore, strengthening the institutional quality and creating a stable political environment would lessen the accumulation of fiscal deficit.

## 1. Introduction

In recent years, fiscal policy has become an integral part of the economic process in developing and developed nations to scale up productivity growth and economic development [1]. In the context of sub-Saharan Africa (hereafter, SSA), fiscal policy often leaned towards budget deficits prompted by government involvement in economic activity and the need to fund public infrastructures. In recent times, fiscal positions in SSA have experienced considerable deterioration. Though the SSA nations benefited greatly from the Highly Indebted Poor Country debt relief initiative 2006, which lessened the debt burden on many countries [2], the debt dynamics witnessed a drastic transformation ever since the 2008 and 2009 economic downturn [3]. Therefore, the debt dynamics in sub-Saharan Africa are largely a reflection of the influence of fresh loans occasioned by rising budget deficits.

Since 2010, the public debt in SSA has nearly tripled. The budgetary consolidation process that several nations in the region began after the conflict hindered the COVID-19 epidemic in Ukraine. While total public debt in SSA stood at US$ 583 billion in 2018, debt to GDP ratio escalated from 24% in 2008 to 59% in 2018, making SSA the fastest-growing debt accumulation region. Similarly, the fiscal balance in SSA countries worsened from a surplus of 2.6% in 2008 to a 4.6% deficit in 2019 [4]. As nations increasingly turned to strategies including subsidies, temporary exemptions of tariffs and levies, and income support for the most vulnerable individuals, the region’s fiscal deficit expanded to 5.2% of GDP in 2022 from 4.8% in 2021 [5].

However, despite the rapid surge in SSA’s fiscal deficit, the affairs of the countries in the region exhibit a paradox of sorts. While traditional wisdom postulated that deficit financing accelerates the growth and development of an economy, the sub-Saharan African region seemed to have an increasing number of poor people than the rest of the world [6]. With mounting fiscal deficits and worsening development indicators, an appropriate question is: what is driving the persistent increase in the budget deficit in sub-Saharan Africa?

Empirically, a plethora of studies have examined the determinants of fiscal deficit, but results are mixed [7–12]. Still, most extant studies on sub-Saharan Africa have wholly focused on the economic determinants of fiscal deficit [1, 13, 14]. These studies presume that high fiscal deficits are purely driven by macroeconomic factors arising from undesirable consequences of economic shocks. This might be too restrictive in empirical analysis. Theoretically, budget deficits ought to diminish during the economic boom; evidence has shown the contrary, where deficits persist to increase even after prosperity in many developing countries [15].

With the advent of political economics, it has been recognised that political factors could be an essential driver of fiscal deficit. Economic theory cannot illustrate the rising fiscal deficit alone; institutional and political processes are an equally vital determining factor of fiscal deficit [11, 16–19]. For instance, [20] argued that macroeconomic factors may not be the only determinant of deficit spending, given that countries facing similar economic shocks have exhibited notable heterogeneity and variations in the magnitude of their fiscal deficit. Thus, institutional and political factors are partly responsible for the sustained rise of budget deficits across countries. Most importantly, given the increasing role of government in modern economies, institutional and political factors could shape the propensity to engage in deficit financing, especially in less developed countries like SSA that are characterised by low capital formation arising from low-income and high consumption propensity.

Therefore, the importance of fiscal policy and the persistence of fiscal deficits in SSA have raised some critical issues about the determinants of fiscal deficit among SSA countries. Notably, what are the drivers of fiscal deficits in sub-Saharan Africa? Are fiscal deficits explained by a set of economic indicators, or do political and institutional factors bias fiscal policy in the direction of deficit spending? These questions have not been addressed sufficiently in the context of sub-Saharan African countries with a persistent rise in fiscal deficit over the years. Hence, examining the determinants of fiscal deficits is crucial, given that the continuous incurrence of fiscal deficits is fast leading to a high surge in debt levels in sub-Saharan African countries [21], which could result in a debt crisis and further exacerbate the already struggling economies in sub-Saharan Africa.

This study contributes to the body of knowledge as follows: First, the study extends extant studies on the determinants of fiscal deficit by including political and institutional factors, largely missing in SSA literature. Besides, choosing SSA as the focus of the study is strategic and appropriate given the rising fiscal deficit. Second, unlike previous studies, this study accounts for cross-sectional dependent and spatial heterogeneity. Globalisation has made SSA share common external shocks arising from trade, capital movement, financial system, etc., which may lead to cross-country dependence [22–24]. Third, the study disentangles the short and long-run dynamics among the variables, which is important for policy prescription.

The study is organised as follows: The theoretical and empirical literature is presented in section 2. The methodology and data are described in section 3. Section 4 provides the results and discussion of findings. Finally, the last section concludes the study.

## 2. Literature review

Different models on the causes of the budget deficit have been developed. These theories include the political budget cycle theory, the Keynesian theory, and the Ricardian theory. The Keynesian hypothesis holds that governments may increase spending during downturns through deficit financing to encourage productivity growth. The public authority may not be able to achieve their desired revenue due to a sharp fall in revenue, which results in a fiscal deficit. This suggests that Keynesian theory holds that economic indicators drive an economy’s fiscal deficit.

On the other hand, the Ricardian hypothesis contends that deficit-financed tax cuts will only shift the burden of paying taxes to future generations. As a result, deficit spending has little impact on an economy. This implies a connection between the current generation and the one to come through benevolence [25].

Nevertheless, Ricardian equivalency questions whether government financing might impact macroeconomic variables like aggregate demand and the current account. The theoretical interpretation is that deficit financing has no long-term influence on macroeconomic results. According to the political budget cycle hypothesis, political conflicts of interest may incentivise elected officials to engage in deficit spending to garner support and win an election. [26], who broke from the equilibrium position to support deficit financing by [25, 27], were the first to offer contributions to this school of thought. They established that differences in political procedures are to blame for various types of fiscal deficits among OECD countries between 1960 and 1985. [28], who advanced the idea that government funding and indebtedness are higher under irregular governments with significant degrees of divergence, are two more major contributors to the political economy school of thought.

On the empirical side, [20] looked at the institutions, politics, and economics as factors of budget deficit volatility in various Association of South East Asian Nations (ASEAN) nations, including Indonesia, Thailand and Vietnam, during the period 1990–2018. Time-series data for each nation were analysed using the fixed effects model (FEM), the random effects model (REM), and the ordinary least squares (OLS). The authors discovered that political stability and corruption are crucial factors influencing the budget deficit. To avoid a big and unstable deficit, it was also advised that greater thought should be given to improving the institutional framework of the economy.

[29] investigated the impact of budget balances, during the Covid-19 pandemic, on 43 different countries. The system generalised technique of moments procedure was used in the investigation. The findings demonstrated that the worldwide pandemic caused an unjustified increase in the magnitude of estimated impacts of the macroeconomic variables employed as drivers on the overall effect on the budget balance.

[7] examined the factors influencing the primary budget balance in a group of 27 European Union member states using panel data methodology. The authors were primarily concerned with determining the significance of fiscal laws, fiscal councils, governance, and the effects of electoral pressures. Still, they also considered other macroeconomic factors like debt, GDP growth, and unemployment rate. They discovered that increasing debt cuts deficits and balances the budget. They contend that greater unemployment rates and election years lead to larger deficit expenditures. Additionally, they demonstrate how the presence of fiscal regulations dramatically lowers deficits. However, they do not discover any appreciable impacts of GDP growth, bond rates, or political leaning on the budget balance.

Additionally, an unbalanced panel of developed and transitional countries encompassing the period 1980 and 2014 was analysed by [30] to determine factors and the effects of fiscal counter-cyclicality. The major findings are that fiscal counter-cyclicality has a clear correlation with political issues, economic development, trade openness, government size, and financial deepening. The relationship between budget deficit and a number of macroeconomic variables, including the gross domestic product, trade balance, inflation rate, unemployment rate, and current account over the period 2000–2018, was analysed by [31]. The author used ARMA and conventional least squares techniques. A long-term co-integration relationship between deficit and macroeconomic indicators was discovered. While the current account and inflation rate had a favourable impact on the budget deficit, gross domestic product, the balance of trade, and the unemployment rate had a negative impact.

The goal of [32] was to identify the economic drivers of fiscal deficit in the general budget of the Palestinian Authority from 1995 to 2013. The current account ratio to GDP, the rate of investment, the unemployment rate, the rate of inflation, the price of foreign exchange, as well as political variables were all chosen as independent variables in the study. The deficit before grants and aid to GDP was used as the dependent variable, and the quantitative standard method was used to build the standard model. The analysis discovered a statistically significant positive correlation between each independent variable and GDP but a substantial negative correlation between GDP current account ratio, inflation rate, and foreign exchange rate.

In their 2010 study, [9] looked at the effects of extreme weather on budget balances in developing nations, OECD nations, and EU nations. Results accounting for macroeconomic, financial, and political factors reveal that each nation group has a different fiscal deficit response to weather shocks. They demonstrate how severe welther incidents impact budget balances in emerging nations with nascent democracies and poor institutions. The findings also indicate that real GDP growth, inflation, and delayed changes in the debt ratio were statistically significant and positively correlated with budget balances. Further results suggest that the delayed change in the nominal long-term interest and election year dummy for the OECD and EU nations reveals a negative coefficient. In their analysis of public spending in 33 parliamentary democracies between 1972 and 2000, [33] found that single-party governments are more likely to alter the budget in accordance with the state of the economy, allowing them to increase or decrease spending with greater flexibility than coalition governments. According to the authors, because each coalition member has veto power, it is challenging to change expenditures when there are challenging fiscal conditions.

[34] examined the political, institutional, and economic causes of public deficit volatility between 1980 and 2006 using a sample of 125 countries via system-GMM estimation for linear dynamic panel data models. They presented three key findings: first, democracy reduces the volatility of public debt; second, political instability is positively correlated with higher levels of volatility of the public debt; third, hyperinflation and trade openness tend to magnify the volatility of the budget deficit, with these effects being powerful for small countries. In their 2007 study, [35] examined the factors that affected fiscal balances in 22 OECD nations between 1970 and 2002. Results showed that changes in budget balances are influenced by changes in debt growth, macroeconomic developments, and political factors and that budget balances significantly worsen in election years. This was evident when looking at the issue from a broad perspective, including the countries, the range of potential explanatory variables, and the time covered.

Additionally, [36] conducted an empirical analysis of the political, institutional, and economic variables that affect the budget deficit for the 15 nations that make up the European Union from 1971 to 2006. The impact of political and institutional factors, unemployment rate, GDP growth rate, cost of debt payment, and other variables are examined using panel-corrected standard errors (PCSE) and fixed effect approaches. According to the findings, the budget deficit strongly correlates with the unemployment rate and the cost of debt service. While government fragmentation and the ideology index have little bearing on the budget deficit, the Maastricht Treaty has a significant impact on the budget deficit, indicating a decline in deficit in European countries.

From 1980 to 2010, [13] looked at the economic factors influencing budget deficits in South Africa. The writers make an apparent effort to determine whether budget deficits result from South Africa’s fight against economic hardships. The authors used the Vector Error Correction Model (VECM) to determine how certain macroeconomic factors affected the budget deficits in South Africa. Except for external debt, it was discovered that all macroeconomic variables had a favourable impact on budget deficits. However, foreign reserves were the primary variable that affected the budget deficit, followed by external debt, unemployment, economic growth, and public investment, in that order.

In related research, [8] investigated the relationship among exchange rate, inflation rate, unemployment rate, gross fixed capital creation and fiscal deficits in Nigeria from 1981 to 2013. The vector error correction mechanism (VECM) was used in the study as an estimating method. The results demonstrated that a high jobless rate reduces budgetary deficits. This implies that budget deficits are increased by policies that seek to create jobs through higher levels of productive investment. The study also shows that rising spending on infrastructure development causes budgetary deficits. However, the results imply a significant negative impact on fiscal deficits.

Additionally, [37] investigated the political and economic factors that influence the durability of fiscal policies in 14 West African nations. The degree to which the government’s current fiscal (revenue and spending) behaviour is linked to its historical behaviour is known as fiscal persistence. The study’s findings demonstrated that government spending, corruption, efficacy, and the rule of Law influence fiscal persistence. [38] conducted a similar investigation on the impact of political factors on Pakistan’s budget deficits. It was determined that the government’s size positively impacts deficits, implying that a large government results in a large budget deficit. The results also demonstrated that weak democratic institutions and low output levels are major contributors to budget deficits.

[14] investigated the Nigerian budget deficit from 1981 to 2016. Johansen co-integration and the Vector Error Correction Model (VECM) technique were employed in the investigation—the results of the Johansen co-integration test point to a sustained relationship between the variables. Further research found that the leading causes of fiscal deficits are interest rates, currency exchange rates, and preceding fiscal deficits.

The aforementioned research makes it clear that no known study has looked at the institutional and political causes of the fiscal imbalance in SSA in addition to the economic causes. By identifying the link between different institutional and political variables on the fiscal deficit in the SSA area, which is recognised as one of the most indebted regions globally, this study addressed the apparent gaps in the literature.

## 3. Theoretical framework, model specification and data

### 3.1 Theoretical framework

This study is based on the Keynesian and the political budget cycle theories. The Keynesian theory holds that fiscal deficit occurs due to an increase in government spending to support productivity growth during the depression. In this view, the Keynesian theory suggests that economic indicators determine a nation’s fiscal deficit. In the political budget cycle theory, elected officials, driven by their political interests, may be motivated to engage in deficit spending as a strategy to gain support and secure victory in an election [26, 28].

### 3.2 Model specification and data

This study examines the drivers of fiscal deficit in sub-Saharan Africa from a multidimensional perspective. Following the theoretical framework, the general model for investigating these determinants is expressed as follows:

$${FD}_{it}=\propto +\beta {ECN}_{it}+\gamma {INS}_{it}+\delta Z_{it}+\varepsilon_{it}$$ (1)

Where i and t denote country i at time t. FD is fiscal deficit; ECN is a vector of economic variables, including per capita income (GDPC), inflation rate (INF) and trade openness (TOP). INS is institutional and political variables which are proxy by one of the following: Corruption (COR), Bureaucracy Quality (BUR), Democratic accountability (DEM), Government stability (GOVS), Internal conflict (ICON), Law and order (LAW), Military in politics (MILP) and Religion tension (REL). Z is the control variable, represented by population (POP). These drivers of fiscal deficit are selected based on extant studies in the literature [7, 39]. However, it is worth mentioning that some new drivers, such as internal conflict, military in politics and religious tension, are introduced due to the role they could play in influencing the extent of public debt in Africa.

This study uses the natural logarithmic of per capita income for income level. Variables such as GDPC, POP, INF and TOP are sourced from World Development Indicators. FD is sourced from international Financial Statistics Data (IMF). All the institutional variables are extracted from International Country Risk Guide (ICRG) data set. The data source and measurement are reported in Table 1. This study works with the most recent available database. Due to unavailable updated data on institutional and political variables, the scope of the study spans from 2000–2018 for twenty-three SSA countries. The list of the countries is presented in S1 Appendix.

**Table 1:** Variables description and characteristic.

Variables	Symbol	Definition	Source	Mean	Median	Max	Min	Std. Dev.	Skewness	Kurtosis	Obs
Fiscal Deficit	FD	Fiscal deficit (% of GDP)	IMF	-1.481	-2.236	31.045	-17.805	5.321	2.246	12.113	475
Per capita income	GDPC	GDP per capita (current US$)	WDI	1748.718	851.421	10809.680	197.833	2136.468	2.190	6.963	475
Inflation	INF	Inflation, consumer prices (annual %)	WDI	8.397	5.161	324.997	-3.503	19.937	11.544	168.555	475
Trade openness	TOP	Trade Openness is the sum of imports and exports normalised by GDP	WDI	63.588	56.655	156.862	20.723	25.786	1.069	3.844	475
population	POP	Population growth (annual %)	WDI	2.629	2.663	5.264	-1.409	0.703	-0.759	7.362	475
Corruption	COR	Index for Control of Corruption	ICRG	2.025	2.000	4.000	0.375	0.665	0.474	3.519	475
Bureaucracy Quality	BUR	index for Bureaucracy Quality	ICRG	1.313	1.479	2.500	0.000	0.661	-0.473	2.590	475
Democratic accountability	DEM	Index of Democratic Accountability	ICRG	3.181	3.000	5.633	1.000	1.031	0.303	1.976	475
Government stability	GOVS	Index of government stability	ICRG	8.257	8.000	11.000	4.458	1.525	0.166	1.852	475
Internal conflict	ICON	Index of Internal Conflict	ICRG	8.415	8.500	12.000	4.583	1.368	-0.104	2.448	475
Law and order	LAW	Index of Law and Order	ICRG	3.016	3.000	5.500	1.000	0.922	0.709	3.133	475
Military in politics	MILP	Index of Military in Politics	ICRG	2.718	2.000	6.000	0.000	1.528	0.292	2.306	475
Religion tension	REL	Index of Religion tension	ICRG	4.015	4.500	6.000	0.500	1.263	-0.506	2.103	475

Where WDI is World Development Indicators, IMF World Economic Outlook, and ICRG is International Country Risk Guide.

In the presence of CD, the Panel Spatial Consistent Correlation estimation (PSCC) technique is used as the baseline. This technique addresses the problems of CD and heteroscedasticity in the panel; also, standard errors are robust to cross-sectional and temporal dependence commonly found in panel data analysis. Consequently, Eq (1) can be specified as:

$${FD}_{it}={X^{'}\theta }_{it}+\varepsilon_{it}$$ (2)

X is the vector of independent variables with (k+1)× vector. The square root of the diagonal elements is used to compute the standard error. The asymptotic covariance matrix of the standard errors could be specified as

$$V\left(\tilde{\theta }\right)={\left(XX\right)}^{-1}\hat{ST}{\left(XX\right)}^{-1}$$ (3)

where $\tilde{\theta }$ is the coefficient estimate, *ST* is the square root and is the vector of independent variables.

Further, Autoregressive Distributed Lag (ARDL) for panel data is used to examine the long-run relationship. Panel ARDL is appropriate in the presence of co-integration and likelihood of endogeneity in the variables. The ARDL provides short- and long-term estimates and the co-integration relationship. Furthermore, the ARDL address the issue of strict exogeneity in the panel dynamics. Because the likelihood of country fixed and time effect influence the result estimates, the dynamic fixed effect estimator is used for the ARDL (DFE-ARDL). The ARDL model for the short and long-run of Eq (1) could be expressed as:

$$\begin{array}{ll} FD_{it}=\propto & +\beta ECN_{it-1}+\gamma INS_{it-1}+\delta Z_{it-1}+\overset{p}{\underset{j=1}{\sum }}\rho_{j}\;\Delta \;FD_{it-j}+\overset{p}{\underset{j=0}{\sum }}\pi_{j}\;\Delta \;ECN_{it-j}\\ & +\sum_{j=0}^{p}\vartheta_{j}\Delta INS_{it-1}+\sum_{j=0}^{p}\chi_{j}\Delta Z_{it-j}+\varepsilon_{it} \end{array}$$ (4)

where β, γ and δ are the long-run parameters, ρ, π, ϑ and χ are the short-run parameters.

## 4. Results and discussion

The descriptive statistics of the series in Table 1 hints that all the variables are normally distributed since the measures of central tendency are very close. Further, the descriptive statistics reveal that the average value of fiscal deficit in the region stood at -1.481 as a percentage of GDP while the maximum and minimum values of fiscal deficit are 31.045 and -17.805, respectively. The average value of per capita income in the region is $1748.718, which is higher than the median value of $851.421. This indicates that per capita income is skewed to the right, and most countries’ income is lower than the mean value.

Inflation, trade openness and population growth rate have an average of 8.397, 63.588 and 2.629, respectively. Furthermore, for institutional variables, the average value of 2.025, 1.313, 3.181, 8.257, 8.415, 3.016, 2.718 and 4.015 was recorded for corruption, bureaucracy quality, democratic accountability, government stability, internal conflict, Law and order, military in politics and religion tension respectively. Apart from this, the skewness statistics hint that all the variables are positively skewed except population, bureaucracy quality, internal conflict and religious tension. Besides, the result from the kurtosis statistic suggests that fiscal deficit, per capita income, inflation, trade openness, population, corruption and rule of Law are leptokurtic since their values are higher than three; hence their distribution is peaked. On the other hand, however, other series included are platykurtic since their values are less than two. The pairwise correlation matrix between the variables is presented in Table 2. The result suggests the absence of multicollinearity among the variables.

**Table 2:** Correlation matrix.

**Correlation**		GDPC	INF	TOP	POP	COR	BUR	DEM	GOVS	ICON	LAW	MILP	REL
**Probability**	FD												
**FD**	1.000												
**GDPC**	0.173^a^	1.000											
	0.001												
**INF**	0.021	-0.092^c^	1.000										
	0.681	0.065											
**TOP**	0.180^a^	0.271^a^	0.272^a^	1.000									
	0.000	0.000	0.000										
**POP**	0.051	-0.437^a^	0.100^b^	-0.117^b^	1.000								
	0.303	0.000	0.045	0.019									
**COR**	-0.016	0.267^c^	0.009	0.187^c^	-0.176^c^	1.000							
	0.751	0.000	0.850	0.000	0.000								
**BUR**	-0.063	0.233^a^	0.044	-0.039	-0.108^b^	0.138^a^	1.000						
	0.209	0.000	0.374	0.436	0.031	0.006							
**DEM**	-0.071	0.135^a^	0.001	-0.164^a^	-0.103^b^	0.192^a^	0.067	1.000					
	0.153	0.007	0.990	0.001	0.038	0.000	0.178						
**GOVS**	0.285^a^	-0.008	0.106^b^	0.267^a^	0.055	0.266^a^	-0.131^a^	-0.029	1.000				
	0.000	0.877	0.034	0.000	0.272	0.000	0.009	0.557					
**ICON**	0.079	0.228^a^	-0.131^a^	0.238^a^	-0.151^a^	0.402^a^	0.015	0.339^a^	0.227^a^	1.000			
	0.113	0.000	0.009	0.000	0.003	0.000	0.762	0.000	0.000				
**LAW**	-0.104^b^	-0.089^c^	0.041	-0.168^a^	0.044	0.116^b^	0.049	-0.011	0.012	-0.038	1.000		
	0.037	0.076	0.415	0.001	0.380	0.020	0.324	0.831	0.813	0.446			
**MILP**	-0.139^a^	0.279^a^	-0.035	-0.158^a^	-0.193^a^	0.364^a^	0.402^a^	0.490^a^	-0.063	0.382^a^	0.172^a^	1.000	
	0.005	0.000	0.487	0.001	0.000	0.000	0.000	0.000	0.204	0.000	0.001		
**REL**	-0.073	0.232^a^	0.031	0.239^a^	-0.332^a^	0.302^a^	0.186^a^	0.157^a^	0.034	0.522^a^	0.094c	0.309^a^	1.000
	0.143	0.000	0.538	0.000	0.000	0.000	0.000	0.002	0.491	0.000	0.060	0.000	

^a^, ^b^ and ^c^ represent 1%, 5%, and 10% significant levels, respectively

Various estimation techniques have been used in the literature to investigate the link among institutional factors, economic variables and fiscal deficit. This study examines the presence or otherwise of cross-sectional dependency (CD) in the panel. The presence of CD affects the true parameters of the estimate if not taken care of in the estimation techniques. Happenings in the world and the interdependence among economies could be responsible for the CD in economic variables [22, 40].

Thus, the presence of CD in the variables is checked using Breusch-Pagan LM, Pesaran Scaled LM, Bias-corrected Scaled, and Pesaran CD tests. The result of the CD tests in Table 3 established the existence of CD in all variables. Hence, the presence of CD in the variables is accounted for in all the estimation techniques used in this study. Furthermore, the second-generation unit root tests in Table 3 hint that all the variables are stationary. Similarly, the result of the slope homogeneity (delta test) in Table 4 established the existence of heterogeneity in the data. The result implies that the slope and coefficients of the model varies across cross sectional unit [41].

**Table 3:** Cross-sectional dependence test and stationary test.

	Cross-sectional Dependence				Unit Root			
Variables	Breusch-Pagan LM	Pesaran Scaled LM	Bias-corrected Scaled	Pesaran CD	CADF Level	CADF 1^st^ Difference	CIPS Level	CIPS 1^st^ Difference
FD	994.060^a^	27.314^a^	26.619^a^	17.780^a^	2.878	-10.437^a^	-2.011	-2.963^a^
GDPC	4785.925^a^	182.116^a^	181.422^a^	68.325^a^	-1.379	-2.694^a^	-1.488	-2.910^c^
INF	718.548^a^	16.066^a^	15.372^a^	10.622^a^	0.058	-2.551^a^	-2.062	-3.160^a^
TOP	913.433^a^	28.337^a^	27.698^a^	7.151^a^	0.781	-3.260^a^	-1.935	-4.041^a^
POP	1693.776^a^	55.880^a^	55.185^a^	5.888^a^	-1.386	-2.906^a^	-2.286^b^	-2.856^a^
COR	1292.309^a^	39.490^a^	38.795^a^	2.120^b^	-1.577	-2.719^a^	-2.305	-4.046^a^
BUR	342.155^a^	27.402^a^	28.139^a^	17.121^a^	1.331	-1.992^c^	-0.838	-2.732^b^
DEM	1491.423^a^	51.231^a^	43.902^a^	24.201^a^	-1.612	-3.182^a^	-2.044	-3.161^a^
GOVS	1134.01^a^	296.534^a^	302.753^a^	89.131^a^	-0.047	-3.125	NA	NA
ICON	453.679^a^	53.154^a^	41.468^a^	19.032^a^	-1.880	-2.408^a^	-2.512^a^	-4.212^a^
LAW	1321.173^a^	210.324^a^	98.710^a^	32.164^a^	-0.789	-2.177^c^	-0.785	-2.313b
MILP	438.405^a^	390.857^a^	201.272^a^	92.132^a^	-0.329	-2.102^c^	-0.544	-2.339^b^
REL	2115.32^a^	280.432^a^	268.390^a^	133.412^a^	-0.458	-2.295^c^	-1.711	-2.467^c^

^a^, ^b^ and ^c^ represent 1%, 5%, and 10% significant levels, respectively

**Table 4:** Slope homogeneity tests.

T-statistics	Value	P-value
Corruption model		
Δ	4.789^a^	0.000
Δ_adj_	6.026^a^	0.000
Bureaucracy Quality model		
Δ	3.857^a^	0.000
Δ_adj_	4.853^a^	0.000
Democratic accountability model		
Δ	4.834^a^	0.000
Δ_adj_	6.083^a^	0.000
Government Stability model		
Δ	3.751^a^	0.000
Δ_adj_	4.727^a^	0.000
Internal Conflict model		
Δ	3.386^a^	0.001
Δ_adj_	4.260^a^	0.000
Law and Order model		
Δ	5.283^a^	0.000
Δ_adj_	6.648	0.000
Military in politics model		
Δ	3.784^a^	0.000
Δ_adj_	4.762^a^	0.000
Religion Tension model		
Δ	4.142^a^	0.000
Δ_adj_	5.212^a^	0.000

Where a is 1% level of significant

Eq (1) is also estimated using Feasible Generalised Least Square (FGLS) for robustness check. As part of the methodology contribution of this study to the extant literature, the short-run and long-run effects of the economic and institutional variables are examined. However, the study initially tests for the long relationship among the variables using an approach that is robust to the CD- Westerlund co-integration test. The Westerlund co-integration test is reported in Table 5. In Table 5, individual measure of institutions is used with other explanatory variables. The results of the long-run co-integration test reject the assumption of no long-run relationship among the variables. Specifically, group mean (Gt) and panel mean (Pt) reject the hypothesis of no long-run co-integration in all the models.

**Table 5:** Westerlund Co-integration test.

Statistics	Value	Z-value	P-value
*Co-integration test for f (FD*, *GDPC*, *INF*, *TOP*, *POP*, *INS)*			
When INS = COR			
Gt	-3.209^a^	-4.763	0.000
Ga	-5.047	4.171	0.910
Pt	-16.224^a^	-5.892	0.000
Pa	-6.534	0.903	0.817
When INS = BUR			
Gt	-3.218^a^	-4.806	0.000
Ga	-5.170	4.093	0.932
Pt	-16.057^a^	-5.755	0.000
Pa	-6.289	1.051	0.853
When INS = DEM			
Gt	-2.992^a^	-3.740	0.000
Ga	-4.386	4.583	0.967
Pt	-14.165^a^	-4.207	0.000
Pa	-4.921	1.876	0.970
When INS = GOVS			
Gt	-3.046^a^	-3.994	0.000
Ga	-4.618	4.438	0.997
Pt	-14.510^a^	-4.490	0.000
Pa	-5.892	1.290	0.902
When INS = ICON			
Gt	-2.875^a^	-3.190	0.001
Ga	-4.128	4.744	0.975
Pt	-15.416^a^	-5.231	0.000
Pa	-4.832	1.930	0.973
When INS = LAW			
Gt	-3.087^a^	-4.187	0.000
Ga	-5.085	4.146	0.954
Pt	-16.283^a^	-5.940	0.000
Pa	-6.156	1.131	0.871
When INS = MILP			
Gt	-2.793^a^	-2.802	0.003
Ga	-4.263	4.660	1.000
Pt	-13.071^a^	-3.312	0.001
Pa	-5.393	1.591	0.944
When INS = REL			
Gt	-3.028^a^	-2.829	0.002
Ga	-3.377	6.025	0.967
Pt	-12.665^b^	-2.070	0.019
Pa	-4.399	3.072	0.999

Note: Instis proxy of the institution and political variables, which is replaced by Control Corruption (COR), Bureaucracy Quality (BUR), Democratic accountability (DEM), Government Stability (GOVS), Internal Conflict (ICON), Law and Order (LAW), Military in politics (MILP) and Religion Tension (REL) in each of the models.

^a^, ^b^ and ^c^ represent 1%, 5%, and 10% significant levels, respectively

Our estimates on the impact of institutional, political, and economic variables on fiscal deficit in SSA is presented in Tables 6 and 7. While the results of the PSCC are presented in Table 6, the robustness estimate (FGLS) is reported in Table 7. The results obtained from the PSCC are statistically consistent and unbiased with the robustness result. Hence, the result from the PSCC estimates is discussed as follows.

**Table 6:** Result estimates (PSCC).

Dep. Var: FD	Panel Spatial Consistent Correlation estimation							
GDPC	0.001^a^ (3.65)	0.001^a^ (3.57)	0.001^a^ (3.35)	0.001^a^ (3.97)	0.001^a^ (3.16)	0.001^a^ (3.27)	0.006^a^ (4.23)	0.005^a^ (3.47)
INF	->0.004 (-0.31)	-0.003 (-0.20)	-0.004 (-0.34)	-0.004 (-0.33)	-0.004 (-0.30)	-0.003 (-0.30)	-0.003 (-0.23)	-0.005 (-0.40)
TOP	0.032^b^ (2.46)	0.029^b^ (2.24)	0.029^b^ (2.21)	0.017 (1.28)	0.031^b^ (2.19)	0.029^b^ (2.31)	0.024^c^ (1.90)	0.036^b^ (2.56)
POP	1.257^a^ (3.03)	1.114^a^ (2.72)	1.209^a^ (2.93)	0.966^a^ (2.44)	1.175^a^ (2.82)	1.231^a^ (2.91)	1.076^b^ (2.57)	0.987^b^ (2.47)
COR	-0.547 (-1.55)							
BUR		-0.604^b^ (-2.52)						
DEM			-0.267 (-1.35)					
GOVS				-0.791^a^ (-3.83)				
ICON					0.071 (0.30)			
LAW						-0.414^c^ (-1.87)		
MILP							-0.505^b^ (-2.38)	
REL								0.520^a^ (2.96)
C	-6.809^a^ (-4.97)	-6.570^a^ (-4.60)	-6.696^a^ (-4.74)	-12.693^a^ (-7.06)	-8.082^a^ (-4.49)	-6.280^a^ (-5.11)	-5.680^a^ (-3.74)	-5.285 (-4.02)
Obs	437	437	437	437	437	437	437	437
R-sq	0.086	0.087	0.084	0.128	0.081	0.087	0.099	0.095
No of countries	23	23	23	23	23	23	23	23

^a^, ^b^ and ^c^ represent 1%, 5%, and 10% significant levels, respectively

**Table 7:** Sensitivity analysis.

Dep. Var: FD	Feasible Generalised Least Square (FGLS)							
GDPC	0.001^a^ (3.45)	0.001^b^ (2.77)	0.001^a^ (4.36)	0.001^a^ (3.71)	0.001^a^ (3.49)	0.001^a^ (2.99)	0.001^a^ (5.49)	0.001^a^ (3.55)
INF	-0.003 (-0.32)	-0.002 (-0.25)	-0.004 (-0.36)	-0.006 (-0.67)	-0.001 (-0.09)	-0.003 (-0.31)	-0.002 (-0.27)	-0.004 (-0.41)
TOP	0.032^c^ (2.08)	0.029^c^ (1.98)	0.030^c^ (1.95)	0.018 (1.07)	0.029 (1.66)	0.029^c^ (2.09)	0.024^c^ (1.75)	0.036^b^ (2.15)
POP	1.086^b^ (2.58)	0.906^b^ (2.34)	1.126^a^ (2.97)	0.863^b^ (2.62)	0.996^b^ (2.34)	1.044^b^ (2.54)	0.967^b^ (2.37)	0.925^b^ (2.42)
COR	-0.433 (-1.22)							
BUR		-0.693 (-1.16)						
DEM			-0.184 (-0.78)					
GOVS				-0.863^a^ (-3.60)				
ICON					0.246 (0.64)			
LAW						-0.361 (-1.30)		
MILP							-0.497 (-1.58)	
REL								0.459^b^ (2.50)
C	-6.425^a^ (-4.96)	-5.709^a^ (-3.67)	-6.717^a^ (-4.14)	-12.919 (-6.96)	-8.826^a^ (-3.18)	-5.827^a^ (-5.55)	-5.345^a^ (-4.16)	-5.237^a^ (-4.20)
Obs	437	437	437	437	437	437	437	437
R-sq	0.086	0.085	0.084	0.127	0.079	0.086	0.099	0.095
No of countries	23	23	23	23	23	23	23	23

^a^, ^b^ and ^c^ represent 1%, 5%, and 10% significant levels, respectively

Per capita income significantly positively influences fiscal deficit in the region. This implies that an increase in per capita income worsens the fiscal position of SSA. In order words, this result suggests that an increase in income comes with the contraction of more borrowing. This result might not be unconnected with the weak institutional capacity and inefficiencies in revenue collection and management across sub-Saharan African regions which hinder governments’ ability to utilise increased revenues effectively. As a result, the fiscal deficit may rise as the government fails to capture the revenue gains fully. This finding aligns with the theoretical prediction that the higher the per capita income level, the larger the size of the fiscal deficit. Furthermore, the result supports the empirical submissions of [16, 17]. This, however, is against the submission of [7], who found an insignificant effect of GDP on fiscal deficit.

Conversely, inflation has a negative impact on fiscal deficit, though the effect is not significant in all the models. Trade openness and population growth rate have a significant positive impact on fiscal deficit. This result is not surprising since developing countries like SSA are import-dependent nations which worsens their balance of payment account and subsequently affects their fiscal account. Furthermore, trade openness increases a country’s exposure to external shocks, such as trade instability, resulting in an adverse fiscal position. [42] also found that trade openness increases the fiscal deficit in 66 developing countries. This result is also established by submitting [17, 35] and. The growing population in the region cames with the increase in the cost of providing for the teeming population, resulting in fiscal deficit for most countries. Typically, larger population requires more public services, providing these services to a growing population puts pressure on government budgets and can lead to increased public spending, contributing to fiscal deficits.

The result from institutional and political variables (corruption control, bureaucracy quality, democratic accountability, government stability, internal conflict, Law and order, military in politics, and religious tension) present exciting findings. First, control of corruption and democratic accountability has a negative but insignificant effect on fiscal deficit. This result reveals that the greater the extent of corruption control and public accountability, the lower the value of fiscal deficit. However, the insignificant coefficients hint that the degree of controlling corruption and public accountability in SSA is still very low.

Bureaucracy quality, Law and order, government stability, and military in politics have a significant adverse effect on fiscal deficit, but the significant impact of the rule of Law is not strong. The results imply that bureaucratic quality and the rule of Law are important institutional factors to reduce the fiscal deficit. The military’s involvement in politics also ensures fiscal discipline in the region. Internal conflict and religious tension positively influence fiscal deficit, suggesting that government spends more, especially during crises and war, to procure military goods, which worsens the fiscal account [43]. In addition, during crises, government-generated revenue is always low, thus resulting in a fiscal deficit. This submission aligns with the finding of [44] for 37 African countries. Similarly, as noted by [45], budget deficit persists in the presence of weak institutions.

This study further examines the short and long-run influence of economic, political, and institutional variables on fiscal deficit in SSA. The result of the DFE-ARDL estimates is reported in Table 8. The short and long-run movements’ results also provide some interesting insight into the relationship among the variables.

**Table 8:** Short run and long run estimates.

Dep. Var: FD	DFE ARDL							
Short Run								
EC	-0.730^a^ (-9.00)	-0.672^a^ (-9.38)	-0.667^a^ (-9.42)	-0.688^a^ (-9.88)	-0.689^a^ (-9.34)	-0.675^a^ (-8.83)	-0.706^a^ (-9.66)	-0.699^a^ (-9.71)
D(GDPC)	0.009^a^ (2.63)	0.008^a^ (2.75)	0.008^a^ (2.34)	0.008^b^ (2.54)	0.007^a^ (2.79)	0.009^b^ (2.36)	0.011^b^ (2.40)	0.009^b^ (2.53)
D(INF)	-0.016 (-0.20)	0.023 (0.19)	0.002 (0.02)	0.038 (0.41)	0.007 (0.07)	0.004 (0.05)	.0110 (0.10)	-0.032 (-0.56)
D(TOP)	-0.054 (-1.51)	-0.071 (-1.63)	-0.018 (-0.58)	-0.036 (-1.18)	-0.059 (-1.47)	-0.046 (-1.45)	-0.051 (-1.53)	-0.058 (-1.62)
D(POP)	0.486^a^ (2.63)	0.326^b^ (2.51)	0.419^a^ (2.63)	0.993^b^ (2.55)	0.480^b^ (2.41)	0.482^a^ (2.60)	0.978 (2.56)	0.545^b^ (2.47)
D(COR)	4.339^c^ (1.69)							
D(BUR)		-2.003 (-0.99)						
D(DEM)			0.974 (1.07)					
D(GOVS)				0.326 (1.02)				
D(ICON)					-0.336 (-0.99)			
D(LAW)						1.117 (0.41)		
D(MILP)							0.648 (0.47)	
(REL)								0.895 (0.79)
C	8.044^a^ (8.649)	4.744^a^ (10.61)	2.303^a^ (6.49)	-6.584^a^ (-7.54)	1.489^a^ (3.94)	1.021^b^ (2.35)	2.765^a^ (7.80)	0.935^b^ (2.10)
Long Run Estimates								
GDPC	-0.001^c^ (-1.76)	-0.001^c^ (-1.67)	-0.001^b^ (-2.50)	-0.001 (-0.35)	-0.001^b^ (-2.04)	-0.001^b^ (-2.27)	-0.001^a^ (-2.67)	-0.001^a^ (-3.48)
INF	-0.065^a^ (-2.77)	-0.070^a^ (-2.74)	-0.685^a^ (-3.00)	-0.024 (-1.52)	-0.073^a^ (-2.82)	-0.081^a^ (-3.39)	-0.031 (-1.44)	-0.044^b^ (-2.19)
TOP	-0.097^a^ (-8.34)	-0.106^a^ (-7.84)	-0.019 (-1.58)	-0.065^a^ (-6.86)	-0.086^a^ (-6.35)	-0.091^a^ (-7.35)	-0.091^a^ (-8.19)	-0.111^a^ (-10.04)
POP	1.335^c^ (1.84)	-0.014 (-0.02)	-0.284 (-0.41)	2.384^a^ (5.00)	0.123 (0.15)	1.332^b^ (2.03)	0.392 (0.50)	0.433 (0.65)
COR	0.311 (1.49)							
BUR		-1.668 (-1.45)						
DEM			-0.796^a^ (-4.07)					
GOVS				-0.646^a^ (-6.39)				
ICON					0.168 (0.69)			
LAW						-0.175 (-0.76)		
MILP							-0.417^c^ (-1.76)	
REL								0.791^a^ (3.10)
Obs	414	414	414	414	414	414	414	414

EC is cointegrating equation,

^a^, ^b^ and ^c^ represent 1%, 5%, and 10% significant levels, respectively

Per capita income exhibits different impacts on fiscal deficit. In the short-run per capita income significantly positively influence fiscal deficit while the effect turns negative in the long-run. The result suggests that an increase in per capita income, in the long run, strengthens fiscal position. Accumulation of fiscal deficit in the short run could be used to stimulate consumption and income, saving, and capital formation, which will translate to long-term economic growth and offset the fiscal deficit [46]. The Keynesian proposition also sheds light on the transmission mechanism between fiscal deficit and growth whereby the government increases its expenditure, especially during the recession, to stimulate growth.

The effect of inflation on fiscal deficit is significantly negative in the long run, signifying that inflation reduces the fiscal deficit in SSA. However, the impact of inflation is insignificant in the short run. These results support evidence of time lag in the effect of inflation on fiscal policy. In the long run, trade openness has a significant negative influence on fiscal deficit; however; the impact is not significant in the short-run. This implies that trade openness strengthens the long-run fiscal position of SSA countries. On the other hand, the population growth rate contributes to the increase in fiscal deficit in both periods. This is in tandem with the result obtained from PSCC.

The significant positive, though not that strong, of corruption control on fiscal deficit in the short-run implies that control of corruption adds to fiscal deficit. Meanwhile, the long-run influence of corruption control is not significant. All other measures of the institutional indicator have an insignificant influence on fiscal deficit in the short-run. In the long run, democratic accountability and the military in politics have a significant negative influence on fiscal deficit. As a result, an improvement in these institutional factors will reduce the size of the fiscal deficit. Government stability and religious tension increase the fiscal deficit in SSA.

Lastly, the error correction term suggests the model returns to the long-run equilibrium after short-run distraction. Specifically, the error correction term is negatively signed and significant in all the models with an average value of -0.689.

## 5. Conclusion

By examining the impact of institutional, political, and economic factors on fiscal deficit from several angles, this research adds to the body of fiscal literature. The research looks at 23 sub-Saharan African nations’ per capita income, inflation, trade openness, and population growth from 2000 to 2018. It also looks at eight institutional and political issues. The estimate methods include panel spatial consistent correlation and practicable generalised least squares. The dynamic fixed effect autoregressive distributed lags are used in the study to examine further the impact of institutional and economic elements over the short and long-term.

The results of this empirical study are as follows. First, the study determined that cross-sectional dependency existed in the panel, requiring the employment of CD-resistant procedures. Second, the PSCC and FGLS results show that the magnitude of the budget deficit is decreased by military involvement in politics, bureaucratic quality, government stability, Law and order, and per capita income, as well as by trade openness, population, and religious conflict. Internal conflict and democratic accountability, however, have lower statistical support. Third, the study found evidence that institutional issues, economic variables, and the budget deficit in SSA were long-term co-integrators. Fourth, per capita income has a big short-term good but long-term negative impact. While the short-term factors are insignificant, the long-term effects of inflation on the fiscal deficit are significantly negative. Both periods’ budgetary deficits are positively impacted by population and religious turmoil. This may not be unconnected to the rising population and religious tensions in many SSA countries. Religious turmoil is associated with economic instability, hinder investment, and deter business activities, leading to reduced revenue collection and fiscal imbalances. Similarly, only the reduction of corruption has a short-term positive coefficient of significance on the budget deficit. However, the long-term fiscal imbalance is significantly impacted negatively by democratic accountability, stable governments, and military involvement in politics.

These findings have some significant policy repercussions. First, long-term per capita income increases can slow the budget deficit’s growth in developing nations. Our results provide proof of this. Although the short-term effect worsens the fiscal situation through debt accumulation, an increase in per capita income has the propensity to boost overall production and productive investment over the long term. To raise productivity, governments are recommended to strengthen assistance to the real economy. Additionally, democratic accountability and political stability must be improved in these nations. A more democratic environment will reduce budget deficits by enhancing investor confidence, increasing productivity, and fostering economic development.

Furthermore, initiatives and policies that try to boost the economy and reduce budget deficits are weakened by corruption. Therefore, addressing fiscal deficits in sub-Saharan Africa depends on combating corruption. Overall, improving instistutional quality, effective fiscal management, reducing population growth, inclusive economic policies, and measures to address social challenges and conflicts are crucial for mitigating the rising debts and fiscal deficits in SSA countries.

## Supporting information

S1 Appendix (DOCX)
