# Supplementary material for: Fiscal deficit in sub-saharan Africa: A new intuition from the institution and political drivers
Source: PLoS One. 2023 Sep 8;18(9):e0291150. doi: 10.1371/journal.pone.0291150 (PMC10490844; doi:10.1371/journal.pone.0291150)
Supplement: S1 Appendix — (DOCX) [file pone.0291150.s001.docx]

**Appendix**

List of Countries

| Angola | Cote d'Ivoire | Guinea | Niger | Tanzania |
| --- | --- | --- | --- | --- |
| Botswana | Ethiopia | Guinea-Bissau | Nigeria | Togo |
| Burkina Faso | Gabon | Kenya | Senegal | Uganda |
| Cameroon | Gambia | Liberia | Sierra Leone | Zambia |
| Congo Rep. | Ghana | Malawi | South Africa | Zimbabwe |
